# Predictors for the academic success of first-year physiotherapy students at a South African university

**DOI:** 10.4102/sajp.v76i1.1418

**Published:** 2020-07-09

**Authors:** Sfiso Mabizela, Ronel Roos, Hellen Myezwa, Joanne Potterton

**Affiliations:** 1 Centre for Health Science Education, Faculty of Health Sciences, University of the Witwatersrand, Johannesburg, South Africa; 2 Department of Physiotherapy, Faculty of Health Sciences, University of the Witwatersrand, Johannesburg, South Africa; 3 School of Therapeutic Sciences, Faculty of Health Sciences, University of the Witwatersrand, Johannesburg, South Africa

**Keywords:** selection, academic performance, physiotherapy students, predictors, first-year

## Abstract

**Background:**

Numerous factors may influence academic performance and success in undergraduate physiotherapy programmes. Understanding these factors could assist with student selection and design of support structures.

**Objectives:**

The objective of our study was to explore the amount of variance explained by the National Benchmark Test (NBT) and the National Senior Certificate (NSC) in passing the first year of study and to explore the association between the NBT performance bands and first-year progression outcome.

**Method:**

The sample comprised 2013–2017 student cohorts. Hierarchical regression models were used to explore significant predictors for academic success in the first year of study. The chi-square test was used to assess the association between the NBT performance bands and the categorised progression outcome.

**Results:**

The NBT domains explained 22% of the variance, *R*^2^ = 0.229, *F* (3, 212) = 20.97, *p* = 0.000. The four NSC subjects accounted for 20% of the variance. All seven predicting variables contributed to 43% of the variance in the first year of study, *R*^2^ = 0.435, *F* (7, 208) = 27.29, *p* = 0.000. Associations between NBT domains and GPA: quantitative literacy (Φ = 0.27; *p* < 0.000); academic literacy (Φ = 0.22; *p* < 0.000); mathematics (Φ = 0.18; *p* = 0.014).

**Conclusion:**

Academic success is associated with academic factors as measured by the NBT and physical sciences matriculation results.

**Clinical implications:**

Support programmes in the first year of study are needed to improve student performance and success such as additional tutorials and language enrichment programmes.

## Introduction

Recent literature has begun to address the question of academic success and throughput in higher education institutions globally (McMeeken et al. 2008; Ryan et al. 2017) and locally (Amosun et al. 2012; Chetty et al. 2018; Mbambo 2009). Internationally 25% – 40% of students who enter higher education do not graduate and in South Africa this percentage is 55% (Prince 2016). Much literature has shown that school leaving performance and academic aptitude tests like Standard Assessment Tests (SATs) are important predictors of academic performance and success. Some studies have shown that they account collectively for 25% of the variance and individually have a range of variances reported (Soares et al. 2009). Academic selection criteria are not the only predictors of success and student behaviours, such as study habits, skills, attitudes as well as learning strategies require attention for academic success (Galleher, Rundquist & Barker 2012; Kappe & Van der Flier 2012).

In South Africa, five of the nine universities offering Health Sciences programmes use the results of the National Senior Certificate (NSC) and the National Benchmark Test (NBT) outcomes to select students (Van der Merwe et al. 2016). Both of these tests assess student performance but have different complementary assessment criteria (Prince 2016). The NSC is a norm-referenced assessment whilst the NBT is criterion-referenced (Prince 2016). Norm-referenced assessment positions students according to results achieved from a high to lower ranking, compared to a criterion-referenced assessment which places a student in a proficiency category as per the test domain (Prince 2016). Using the NSC as the primary tool for student selection is not recommended considering the wide range of quality of secondary school education across regions and the resources available. Essack, Wedekind and Naidoo (2012) propose that Health Sciences Faculties could use the NSC as a sole selection tool for students who received quality secondary education and whose first language is English.

The performance band categories of a criterion-referenced assessment indicate the student’s preparedness for the demands of higher education and the extent to which the curriculum at university should be responsive to the student admitted (Prince 2016). This assessment can be used as a means of highlighting a student who might be at risk of failing at university. Selection criteria for NSC involves the following: the NBT ratio differs between medical schools across South Africa and, at the University of the Witwatersrand, the contributing ratio for student selection is 50%:50% (Van der Merwe et al. 2016). The 50% comes from the average of five subjects (English, Mathematics, Life Sciences, Physical Sciences, and one subject in which the student has attained the highest mark, excluding Life Orientation). The NBT contributes the other 50%.

The NBT is assessed in three domains namely: academic literacy (NBT AL), quantitative literacy (NBT QL) and mathematics (NBT MAT), and the results are categorised into three specific performance bands that are named proficient, intermediate and basic (Bohlmann et al. 2015). Academic literacy tests language proficiency for academic engagement whilst QL assesses how well the student can engage with problems that require quantitative problem-solving solutions. The NBT MAT component integrates mathematical concepts and the degree to which students can logically deduce conclusions (Prince 2016).

Although the NBT MAT component relates to mathematical concepts, skills acquired with mathematics are transferable to other courses such as chemistry and physics (Bohlmann et al. 2015; Prince 2016). Table 1 explains the proficiency standards for each band of the NBT and recommended support for the students (NBT Project 2016b).

**TABLE 1:** Proficiency standards for academic, quantitative literacy and mathematics (Centre for Educational Testing for Access and Placement).

NBT Band	Domain	%	Recommended programmes
Proficient	NBT MAT	69-100	Performance suggests that academic performance will not be adversely affected in cognate domains. If admitted, students should be placed in regular programmes of study.
	NBT AL	68-100	
	NBT QL	70-100	
Intermediate upper	NBT MAT	52-68	Students are likely to need complementary support (additional tutorials, workshops, augmented courses, language intensive work)
	NBT AL	54-67	
	NBT QL	55-69	
Intermediate lower	NBT MAT	35-51	Students need to be placed in an extended programme.
	NBT AL	40-53	
	NBT QL	40-54	
Basic	NBT MAT	0-34	Test performance reveals serious learning challenges: it is predicted that students will not cope with degree-level study without extensive and long-term support. Institutions admitting students performing at this level would need to provide such support themselves.
	NBT AL	0-39	
	NBT QL	0-39	

*Source*: National Benchmark Test Project, 2016b, *Benchmark levels*, viewed 09 December 2019, from https://nbt.ac.za/content/benchmark-levels

NBT, National Benchmark Test; MAT, Mathematics; AL, academic literacy; QL, quantitative literacy.

Physiotherapy is one of the degrees offered in the Faculty of Health Sciences. Physiotherapy is a profession concerned with optimising the health-related quality of life by influencing the physical, psychological, emotional and social well-being of individuals within the areas of promotion, prevention, treatment, habilitation and rehabilitation (World Confederation of Physical Therapy [WCPT] 2019). Three key elements to the undergraduate physiotherapy curriculum include the content of concepts taught, the student learning process and the sociocultural context in which physiotherapy is practised (Amosun, Maart & Naidoo 2018).

When selecting students, an adequate number of quality candidates are needed to provide healthcare practitioners who are able to practise in the local society which includes rural and under-serviced areas (Van der Merwe et al. 2016). To address historical inequalities with regard to access to entry into health science degrees, faculties across South Africa attempt to select candidates reflecting the demographic profile of the country (Van der Merwe et al. 2016). In line with these sentiments, Amosun et al. (2012) highlighted the strides made towards reaching this goal by reporting the change in the first-year physiotherapy demographic profile at the University of Cape Town (UCT). In 2008 the racial groups consisted of black students (14%), coloured students (46%), Indian/Asian students (2%) and white students (38%) at UCT. By 2010 the cohort composition changed to black students (34%), coloured students (32%), Indian/Asian students (3%) and white students (31%). The proportion of racial groups reflected at an institution may, however, be influenced by the demographic profile of the province and secondly the financial support and bursaries offered to students (Amosun et al. 2012; Van der Merwe et al. 2016). Every year a set number of offers are made to potential students that often exceeds the capacity of an institution, but fewer students take up the said offer and register for the degree (Van der Merwe et al. 2016).

In the past decade, the success rates of first- and second-year students at the University of the Witwatersrand changed. Informal review of student performance showed a consistent 20% dropout rate as students progress from the first year to the fourth year of study, thus prompting the need to examine the criteria used for accessing entrance into the degree. This is undertaken whilst acknowledging other factors that influence academic success. Factors reported in the literature are older age (Gordon & Williams 2010; Utzman, Riddle & Jewell 2007), ethnicity (Amosun et al. 2012; Mbambo 2009; Ryan et al. 2017), having a previous degree (Ryan et al. 2017) and living off campus (Mbambo 2009; Ryan et al. 2017). Other factors include financial capacity (Ryan et al. 2017), physiotherapy being first choice of study (Mbambo 2009), pre-admission academic preparation (higher graduate certificate of education ordinary level examination [O-level GCE] score) (Gordon & Williams 2010), higher NBT and NSC scores (Rankin et al. 2012) as well as the verbal Graduate Record Examination score (GRE) and quantitative GRE score (Utzman et al. 2007).

The two main assessments used to determine entrance into the degree are the NBT and the NSC and their relation to student performance amongst physiotherapy students is unknown. The aim of our study was, therefore, to understand the amount of the variance explained by the NBT and the NSC, in predicting academic success, to identify significant predictors for academic success and to understand the association between the NBT proficiency bands and students’ progression outcome. Academic success in this study was defined as students who passed all of their first-year courses and were permitted to proceed to the second year of study.

## Research methods and design

### Study design and population

A retrospective record review was undertaken. Academic and non-academic admission data were analysed, of cohorts of physiotherapy students registered for first year at the University of the Witwatersrand between 2013 and 2017.

### Data collection

The data were obtained from the Business Intelligence Services (BIS) unit, for research purposes, at the institution. The BIS is the university analytic and institutional research unit that disseminates information to various departments and centres across the university. The non-academic variables collected were gender, race, and place of origin, school quintile and specific cohort year. Public schools in South Africa are divided into quintiles, with quintile one representing the poorest and quintile five the wealthiest schools (Ogbonnaya & Awuah 2019). Quintile one to three schools are non-fee-paying schools and these schools receive more funding from the government compared to quintiles four and five, where parents pay school fees (Ogbonnaya & Awuah 2019). Academic data included the NSC results of English, Mathematics, Life Sciences and Physical Sciences, the NBT results for the three domains (NBT MAT, NBT AL and NBT QL) and the first-year Grade Point Average (GPA). The GPA was calculated using the results from the first-year degree courses. In the first year of study, students are registered for chemistry, physics, introduction to psychology, basic principles of individual and group psychology, introduction to medical sciences and human behavioural sciences and introduction to physiotherapy.

### Data analysis

The Statistical Package for the Social Sciences (SPSS) was used to analyse data. Hierarchical multiple regression was utilised to examine the predictors for success in the first year of study. The sample comprised 332 students (Table 2) of which 270 (81.1%) passed the first year of study. The remaining data about students who failed 37 (11.1%) or cancelled their studies 25 (7.5%) was removed from the regression model. The independent variables were the results of four NSC subjects (English, Mathematics, Life Sciences and Physical Sciences) and the results from three NBT domains (NBT MAT, NBT AL and NBT QL). The outcome variable was the first-year GPA. A total of 54 cases of students were removed during the process of cleaning the data for analysis whilst others have missing data in the variables of interests; thus, the sample was reduced to *n* = 216. The chi-square test was used to explore the association between the NBT proficiency bands and first-year GPA. The NBT domains were categorised into three bands namely proficiency (P), intermediate upper (IU) and intermediate lower (IL). The classification of NBT domains was in accordance with the results that the students obtained in each domain. The first-year progression outcome was categorised into three levels namely passed, failed and cancelled. In line with the secondary aim of the study, the whole sample (*n* = 332) was analysed. A few students had not taken Life Sciences or Physical Sciences at secondary school, and in consequence their data were removed from this analysis. For the NBT MAT, a Fisher exact test was reported, as an assumption of 5 expected counts was not met, for students who passed with proficient results. The IU and IL bands of the NBT AL were combined, as few students were placed at an IL band. Lastly, the IL and basic level for NBT QL were also grouped together.

**TABLE 2:** Demographic profile of the student cohort (2013–2017).

Demographic	Variable	2013		2014		2015		2016		2017		Total	
		*n*	%	*n*	%	*n*	%	*n*	%	*n*	%	*n*	%
Race	Black	17	29	22	33	18	28	33	49	23	30	113	34
	White	31	53	23	35	29	45	24	36	37	49	144	43
	Coloured	5	8	9	14	7	11	1	1	2	3	24	7
	Indian	5	8	11	17	10	16	9	13	14	18	49	15
	Chinese	1	25	1	2	0		0		0		2	1
Gender	Male	12	20	14	21	14	22	21	31	22	28	83	25
	Female	47	80	52	79	50	78	46	69	54	71	249	75
Place of origin	Rural	3	5	5	8	7	11	18	27	10	13	43	13
	Urban	48	81	57	86	57	89	48	72	63	83	273	82
	Unknown	8	14	4	6	0		1	1	3	4	16	5
Residence	Living in university residence	6	10	6	9	13	20	18	27	5	7	48	14
	Private residency	53	90	60	91	51	80	49	73	71	3	284	86
English	English first language	52	88.1	55	55	55	86	42	63	58	76	262	79
	English second language	5	8.5	8	8	8	8	20	30	14	18	55	17
	Missing	2	3.4	3	1	1	1	5	7	4	5	15	5
School quintile	SQ1	0	0	1	2	1	2	6	9	3	4	11	3
	SQ2	2	3	1	2	3	5	4	6	3	4	13	4
	SQ3	1	2	5	8	3	5	7	10	5	7	21	6
	SQ4	4	7	8	12	6	9	3	4	7	9	28	8
	SQ5	44	75	48	73	51	80	46	69	54	71	243	73
	Unknown	8	14	3	5	0	1	1	1	4	5	16	5
First-year outcome	Proceed	54	91	57	86	49	76	47	70.1	63	83	270	81
	Cancelled	1	2	6	9	1	2	11	16.4	6	8	113	34
	Failed	4	7	3	5	14	22	9	13.4	7	9	144	43

SQ, school quintile

School quintile 1 poorest; and School quintile 5 least poor.

The demographic data of students were analysed using frequency tables. Descriptive statistics for continuous variables and the mean and standard deviation were reported. The analyses of student results in the NBT domains were performed. Two regression models were performed and the *R*^2^ coefficient of the two models was compared. In the first models, only the NBT domains were entered in the regression equation. In the second model, the four NSC subjects were added in the model. This made possible the comparison of the amount of variance accounted for by the NBT and the NSC. The ANOVA test was used to assess the predictive utility of the model. The standardised coefficients were assessed to understand the contribution of each independent variable added in the model. The effects size of the model was calculated and reported. In the chi-square test, the Pearson chi-square and the levels of association were reported.

### Ethical considerations

The University of the Witwatersrand Human Research Ethics Committee (HREC) approved the study. The clearance certificate number is: M170490.

## Results

### Demographic profile of the student population

The population consisted of 25% (*n* = 83) male and 75% (*n* = 249) female students (Table 2). Concerning racial groups, most students were white students 43.4% (*n* = 144) or black students 34% (*n* = 113) (Table 2). Most students (82.2%, *n* = 273) were of urban origin compared to rural origin [13% (*n* = 43)] (Table 2). In the light of the students’ place of origin, 73.2% (*n* = 243) attended quintile five schools (Table 2). Few students were living in university residences (14.5%; *n* = 48) whilst most were living in private residences during their studies (85.5%; *n* = 284) (Table 2). In 2013 the highest pass rate into the second year was achieved (91%; *n* = 54) with the lowest being in 2016 (70.1%; *n* = 47). More students cancelled their registration in 2016 (16.4%; *n* = 11). Most students were first language English speakers (79%; *n* = 262).

### Descriptive profile of the academic status

The descriptive profile of the student population concerning academic performance is in Table 3. The student population scored highest in the NBT AL domain (70.41±9.36) achieving a proficient performance band level (Table 3). The first-year GPA was lower than the respective matriculation courses. The students received a lower score in the NBT MAT domain compared to NSC mathematics and physical sciences.

**TABLE 3:** Descriptive profile of academic proficiency levels of cohort (2013–2017).

Academic Parameter	*N*	Mean	SD
NBT Mathematics	216	55.79	13.88
NBT Academic Literacy	216	70.41	9.36
NBT Qualitative Literacy	216	63.61	11.66
English	216	76.39	6.50
Mathematics	216	76.48	7.97
Life Sciences	216	79.69	6.51
Physical Sciences	216	72.97	8.66
First year GPA	216	67.17	6.92

NBT, National Benchmark Test; GPA, Grade Point Average.

The distribution of students as per NBT domains and proficiency bands is in Table 4.

**TABLE 4:** National Benchmark Test domains of the cohort (2013–2017).

Academic Parameter	*N*	Proficiency		Intermediate Upper		Intermediate Lower	
		*n*	%	*n*	%	*n*	%
NBT MAT	329	48	14.5	135	40.7	146	44.0
NBT AL	330	204	61.4	126	38	-	-
NBT QL	330	102	30.7	134	40	94	28.3

NBT, National Benchmark Test; MAT, Mathematics; AL, academic literacy; QL, quantitative literacy.

More of the sample scored in the IL band for NBT MAT (44%; *n* = 146) and in the IU band for NBT QL (40%; *n* = 134) (Table 4). Most students (61.4%; *n* = 204) scored in the proficient band for NBT AL (Table 4).

### Associations and predictors for academic success

The NBT domains accounted for a statistically significant (22%) percentage of variance, *R*^2^ = 0.229, *F* (3, 212) = 20.972, *p* = 0.000. When Life Sciences, English, Mathematics and Physics were added in the regression model, they contributed 20% of the variance, *R*^2^ = 0.206, *F* (4, 208) = 18.93. All predicting variables collectively accounted for 43% of the variance in the first year of study, *R*^2^ = 0.435, *F* (7, 208) = 27.97, *p* = 0.000. The unstandardised (*B*) and standardised (β) regression coefficient and squared semi-partial correlations (*sr*2), for the unique contribution of each predictor, are reported in Table 5. By Cohen’s convention, a combined effect of this magnitude was large (*f*2 = 76).

**TABLE 5:** Regression models of predictors of success (*N* = 216).

Variables	Unstandardised Coefficients		Standardised Coefficients	*sr*^2^
	*B*	95% CI	Beta	
**Model 1**				
NBT MAT	T0.111	0.046, 0.177	0.223	0.001
NBT AL	0.301	0.199, 0.403	0.407	0.000
NBT QL	−0.039	−0.126, 0.049	−0.065	0.385
**Model 2**				
NBT MAT	0.003	−0.066, 0.071	0.005	0.940
NBT AL	0.228 ***	0.132,0.325	0.309	0.000
NBT QL	−0.030	−0.108, 0.047	−0.051	0.439
English	0.204 ***	0.068, 0.340	0.192	0.003
Maths	0.049	−0.083, 0.182	0.057	0.464
Life sciences	−0.045	−0.204, 0.114	−0.042	0.579
Physical sciences	0.329 ***	0.202, 0.455	0.411	0.000

CI, confidence intervals; NBT, National Benchmark Test; MAT, Mathematics; AL, academic literacy; QL, quantitative literacy.

*** , *p* < 0.000.

The associations between the NBT MAT proficiency band and the first-year GPA was statistically significant, Σ^2^ (4, *N* = 329) = 11.80, *p* < 0.014. The association was weak, Φ = 0.18 (Table 6). The associations between the NBT AL proficiency band and the first-year GPA was statistically significant, Σ^2^ (4, *N* = 330) = 16.40, *p* < 0.000. The association was weak, Φ = 0.22 (Table 6). The associations between the NBT QL proficiency band and the first-year GPA was statistically significant, Σ^2^ (4, *N* = 330) = 24.58, *p* < 0.000. The association was weak, Φ = 0.27 (Table 6).

**TABLE 6a:** Associations between National Benchmark Test domains and first year Grade Point Average categories.

Variable	*N* = 329	Proficient *n* = 48 (14.6%)		Intermediate upper *n* = 135 (41%)		Intermediate lower *n* = 146 (44.4%)		*p*	φ
		*n*	%	*n*	%	*n*	%		
**NBT Mathematics**								< 0.014	0.18
Proceed	268	43	16	116	43.3	109	40.7	-	-
Fail	36	1	2.8	10	27.8	25	69.4	-	-
Cancel	25	4	16	9	36	12	48	-	-

NBT, National Benchmark Test.

**TABLE 6b:** Associations between National Benchmark Test domains and first year Grade Point Average categories.

Variable	*N* = 330	Proficient *n* = 204 (61.8%)		Intermediate upper *n* = 126 (38.2%)		Intermediate lower	*p*	Φ
		*n*	%	*n*	%			
**NBT Academic literacy**							< 0.000	0.22
Proceed	269	180	66.9	89	33.1	-	-	-
Fail	25	13	36.1	23	63.9	-	-	-
Cancel	36	11	44	14	56	-	-	-

NBT, National Benchmark Test.

**TABLE 6c:** Associations between National Benchmark Test domains and first year Grade Point Average categories.

Variable	*N* = 330	Proficient *n* = 102 (30%)		Intermediate upper *n* = 134 (40.6%)		Intermediate lower *n* = 94 (28.5%)		*p*	Φ
		*n*	%	*n*	%	*n*	%		
**NBT Quantitative literacy**								< 0.000	0.27
Proceed	267	90	33.5	117	43.5	62	23	-	-
Fail	35	5	13.9	9	25	22	61.1	-	-
Cancel	28	7	28	8	32	10	40	-	-

NBT, National Benchmark Test.

## Discussion

The objective of the study was to explore predictors for academic success in the first year of study of students at the University of the Witwatersrand, and to determine the associations, between the NBT proficiency bands and first-year GPA, of results attained. Academic success in this study amounted to students who passed all of their first-year courses and proceeded to the second year of study. A passing grade is a mark of ≥ 50% for a course.

Eighty-one per cent of the study cohort proceeded into the second year and the failure rate over the years of the review was 11% (*n* = 37). The failure rate per year of study fluctuated (2013 [7%; *n* = 4], 2014 [5%; *n* = 3], 2015 [22%; *n* = 14], 2016 [13.4%; *n* = 9], 2017 [9%; *n* = 7]) but was similar compared to other literature. Ryan et al. (2017) reported a failure rate of 11% (*n* = 38) during a study period of 2010–2013 when reviewing the first-year throughput at an institution in the United Kingdom (UK). The authors reported that multivariate analysis factors associated with dropping out of the degree due to failure were a black or Asian racial grouping. McMeeken et al. (2008) reviewed the failure rate of physiotherapy students over the years of study at five universities across Australia, noting that the first-year failure rate was the highest: first year (11%), second year (6%), third year (3%) and fourth year (1%). When reviewing our study findings, it should be remembered that in 2015 and 2016 the higher education institutions experienced the #FeesMustFall Movement and activity related to this movement escalated during October of each year when examinations historically took place (Langa et al. 2017). Student protests started peacefully but progressively turned more violent in 2016, and that subsequently disrupted processes at respective universities across South Africa (Langa et al. 2017). The higher failure rate seen in the performance of students in 2015 and 2016 in our study might therefore be due to their experiences endured during this period. Aside from the failure rate, a number of students cancelled their registration in the first year (8%; *n* = 25).

Data related to reasons why students cancel their registration in first year is not available from the BIS unit at the institution and is thus not available for review. Ryan et al. (2017) report that a student attaining a B in the A-level Biology course was associated with dropping out, due to voluntary withdrawal in a first-year UK student cohort. Locally, Mbambo (2009) report that reasons for students leaving the physiotherapy programme during the course of their studies at institutions across the country were a change in career choice or frequent failures that resulted in academic exclusion.

Our study highlights the positive associations between the students’ progression outcomes at the end of the first year and the three domains of the NBT, of which the two literacy components had slightly stronger associations than the mathematics component of the NBT. These findings support the use of the NBT as a selection tool in physiotherapy education as a means of predicting academic first-year success. A low score in the NBT AL band will result in students experiencing challenges in areas such as academic reading and reasoning ability, and many students might struggle to engage successfully with the academic demands of higher education (Prince 2016). Predictors for academic success for the first year of study at the presenting institution were the NBT AL, English and Physical Sciences results.

University candidates may write the NBT in English or Afrikaans but the language chosen is influenced by the language policy, of the tertiary institution that the candidate wishes to attend (NBT Project 2016a). The language policy at the University of the Witwatersrand is that all activities related to teaching and learning are in English. Achieving good or excellent results in the NSC English language subject does not automatically result in a proficient placing when writing the NBT (Bohlmann et al. 2015) or relate to success in first year (Mashige, Rampersad & Venkatas 2014). Bohlman et al. (2015) noted that students who achieved a good result (> 80%) in the NSC English Home Language or English First Additional Language had variable NBT AL results. Mbambo (2009), when interviewing heads of departments at physiotherapy programmes across South Africa, identified that one aspect that influenced the success of physiotherapy students is the students’ proficiency in the language of instruction at the institution. The NBT AL and students not being first language English speakers may therefore be a screening method for highlighting students who may be at high risk of not succeeding in their first year of study, and who will require additional support. The nature of academic literacy programmes may need to be generic, for example teaching students to read academic text and write logically, or it may need to be discipline-specific (Butler 2013). Further discourse and research is needed in the physiotherapy education sphere in South Africa to investigate optimal strategies for addressing the current literacy state of the student body.

Most first-year students in this physiotherapy population achieved a less than proficient status as per the NBT MAT domain, highlighting that additional support structures are needed to assist students toward academic success. Bohlmann et al. (2015) reported a correlation of 0.708 between the NBT MAT domain and NSC Physical Science score for the Bachelor degree level candidates of the 2015 matriculation cohort. The authors noted that candidates with an NSC grading > 80% for Physical Sciences had variable NBT MAT results. In addition, NBT MAT results were more in line with trends observed in the pass rate of first-year students concerning courses in mathematics or related disciplines (Bohlman et al. 2015). Our study supports the suggestion of Bohlman et al. as the matriculation results for physical sciences were a predictor for students proceeding into the second year. Students in the intermediate bands or basic bands of the NBT MAT are more likely to experience academic difficulties with subjects such as chemistry and physics due to the nature of these courses where mathematical concepts are used (Prince 2016). The current entry requirements for physiotherapy at the University of the Witwatersrand require a student to have English, Mathematics, Life Science or Physical Sciences as subjects in matric. Students who do not have physical sciences at secondary school have the option to apply for the degree in physiotherapy at the university. Support programmes offered to students concerning physics and chemistry were additional tutoring sessions. These sessions were available to students but were not compulsory. Considering the findings of our study, tutorial sessions may need to become compulsory for students who require such sessions. Future discussion regarding student selection as it pertains to the physical science course component may warrant consideration. In addition, consideration to refer potential students on to other institutions, where physics and chemistry are not part of the curriculum of the physiotherapy degree, when a student did not have physical sciences at secondary school, should be considered.

The demographic profile of the first-year study population highlighted that physiotherapy at the University of the Witwatersrand remains a female-dominated career choice. This is similar to findings reported by other authors in physiotherapy education locally (Amosun et al. 2012; Chetty et al. 2018; Mbambo 2009) and internationally (McMeeken et al. 2008; Ryan et al. 2017). In addition, most students admitted into the programme during the course of the study were white students (43%; *n* = 144) and black students (34%; *n* = 113). Review of yearly data noted a progressive increase in the admission of black students with 2016 student enrolments in these two racial categories being black students (49%; *n* = 33) and white students (36%; *n* = 24). Strides have been made concerning addressing historical inequalities when reviewing our study’s racial findings with data reported by Mbambo (2009). The latter author noted the data of these two groups being black students (11%; *n* = 4) and white students (89%; *n* = 34) in 1994 which changed to black students (31%; *n* = 12) and white students (69%; *n* = 27) in 2005.

Comparing our study’s findings, concerning these two racial categories, indicates data that are similar to those reported by Amosun et al. (2012). Van der Merwe et al. (2016) emphasised that the selection of students into the Health Sciences faculties is also based on the aim to provide practitioners who are able to practise in our local society, such as rural or under-serviced areas. Our student selection process for entry into a Health Sciences degree at the institution was revised in 2014. Since 2015, student selection is now offered to potential candidates as follows: 40% of places are offered to top achievers; 20% of places are offered to rural candidates; 20% are offered to candidates from quintile one and two schools and 20% of places are offered to high achieving black and coloured students (Wits Media 2014). The selection criteria were altered to ensure that the institution is demographically diverse, and an excellent institution supporting a democratic and non-racial South Africa (Wits Media 2014). Considering the revised selection criteria, and the demographic profile of our study, more work needs to be focused on attracting students from rural or quintile one and two schools. As a result of the institution being in an urban city, adequate financial support structures such as student scholarships and residence placements should be in place to attract these students and ensure academic success over the course of their degree.

## Conclusion

Our study demonstrated the benefit of using a criterion assessment in the form of the NBT, as part of the student selection process, into the physiotherapy programme, as it highlighted the associations between the three domains of the NBT and the GPA attained in the first year of study. Our findings, however, suggest that support structures and programmes are necessary as a means to improve the academic performance of first-year physiotherapy students. Such programmes could be offered during available timetabled sessions and consideration should be given to making these sessions compulsory. Other options might be bridging programmes or the possibility of an extended curriculum, which could, however, require additional funding, and a staff complement to ensure success. A limitation of this study is that results are related to one institution in an urban city in South Africa.
